# Ironing Out the Details: How Iron Orchestrates Macrophage Polarization

**DOI:** 10.3389/fimmu.2021.669566

**Published:** 2021-05-12

**Authors:** Yaoyao Xia, Yikun Li, Xiaoyan Wu, Qingzhuo Zhang, Siyuan Chen, Xianyong Ma, Miao Yu

**Affiliations:** ^1^ State Key Laboratory of Livestock and Poultry Breeding, Key Laboratory of Animal Nutrition and Feed Science in South China, Ministry of Agriculture and Rural Affairs, Guangdong Provincial Key Laboratory of Animal Breeding and Nutrition, Guangdong Engineering Technology Research Center of Animal Meat Quality and Safety Control and Evaluation, Institute of Animal Science, Guangdong Academy of Agricultural Sciences, Guangzhou, China; ^2^ College of Animal Science, South China Agricultural University, Guangzhou, China; ^3^ Maoming Branch, Guangdong Laboratory for Lingnan Modern Agriculture, Guangzhou, China

**Keywords:** iron, inflammation, macrophage, metabolism, epigenetics

## Abstract

Iron fine-tunes innate immune responses, including macrophage inflammation. In this review, we summarize the current understanding about the iron in dictating macrophage polarization. Mechanistically, iron orchestrates macrophage polarization through several aspects, including cellular signaling, cellular metabolism, and epigenetic regulation. Therefore, iron modulates the development and progression of multiple macrophage-associated diseases, such as cancer, atherosclerosis, and liver diseases. Collectively, this review highlights the crucial role of iron for macrophage polarization, and indicates the potential application of iron supplementation as an adjuvant therapy in different inflammatory disorders relative to the balance of macrophage polarization.

## Introduction

As an essential microelement, iron is involved in cell proliferation, metabolism and differentiation (1). Generally, recycling iron from red blood cells (RBCs) is essential for maintaining the quantity and function of RBCs. In mammals, approximately 95% of the iron required for physiological processes is dependent on macrophage iron recycling *via* a series of complex processes (2–7). Of note, the roles of macrophages in regulating iron homeostasis have been well addressed (8, 9).

Reciprocally, iron modulates macrophage fate and function, like the development and differentiation of tissue-resident macrophages (10–18), the function of macrophages in hematopoiesis (19–22), and macrophage-mediated inflammatory responses during pathogen infections (23–28). Noteworthily, in addition to their homeostatic activities, macrophages polarize into disparate phenotypes in response to external signals and stimuli to exert different functions (e.g., regulating the progression of diseases) (29). Numerous regulators, including cellular pathway, intracellular metabolism, and epigenetic regulation contribute to the polarization of macrophages, which have been well summarized elsewhere (30–32). Therefore, in this review, we specifically focus on whether and how iron shapes macrophage polarization.

## Regulation of Iron in Macrophage Polarization

It is well accepted that, under normal conditions, iron always binds with ferritin (Ft) in macrophages (33); nevertheless, iron homeostasis and expression of iron-regulated genes strikingly shift during macrophage polarization. For example, in classically-activated macrophages (M1-like, pro-inflammatory phenotype), expression of *Hamp* (encoding for hepcidin) and *FtH*/*FtL* (encoding for ferritin) are highly up-regulated; while *FPN* (encoding for ferroportin) and *IRP1*/*2* (iron regulatory proteins) are down-regulated (34, 35). Such different expression of Fe-regulated genes in polarized macrophages suggests that iron might be associated with macrophage activation.

Notably, acute iron deprivation decreases expression of activating transcription factor 4 (ATF4) in resting human macrophages, and reduces interleukin (IL)-1β and tumor necrosis factor (TNF)-α expression in human macrophages following LPS treatment (36). Moreover, iron depletion blocks NF-κB activation in rat alveolar macrophage stimulated by LPS plus TNF-α (37). These fascinating findings indicate that iron may be necessary for M1-like macrophage polarization. However, more investigations are needed to support this notion as only few studies focusing on the iron deprivation in modulating macrophage activation (**Table 1**). Moreover, the current studies seem to be inclined to investigate the effects of iron depletion on M1-like macrophage polarization; therefore, it is interesting to have a comprehensive understanding about iron deprivation on macrophage polarization [e.g., alternatively-activated macrophage polarization (M2-like, anti-inflammatory phenotype)] in the future.

**Table 1:** Iron affects macrophage phenotypes.

Cell Types	Inducers	Treatments	Changes of mediators	Refs
Human macrophage	LPS (100 ng/ml), 24 h	Deferiprone (DEF) (0.5 mM)	ATF4↓ IL-1β↓ TNF-a ↓ TGF-β↑	(36)
Rat alveolar macrophage	LPS (1 μg/ml)+TNF-α (4 U/ml), 30 min	Iron-deficient diet	NF-κB activity↓	(37)
	LPS (1 μg/ml) + TNF-α (4 U/ml), 30 min	Iron dextran (10 mg/125 mg)	NF-κB activity ↑	(37)
Microglia	Not provided	Endogenous iron absorbed by macrophages	TNF ↑	(38)
Mouse and rat macrophages	LPS (5 μg/ml)+ IFN-γ (100 U/ml)	Fe^2+^ (FeSO_4_, 0.04, 0.2, 1 mM)	NO↓	(39)
PMA-primed THP-1	Resting condition	Hepcidin, 24 h	IFN-γ/IL-4 ratio↑ iNOS↑	(40)
THP-1	Resting condition	FeCl_3_ (0.01 mM), 5 min Hemin (0.01 mM), 5 min	IL-6 ↑	(41)
	LPS+IFN-γ or IL-4+ IL-13, 20 ng/ml, 24 h	FAC (0.1 mM)	IL-1β↓ TNF-α↓ *CCL17*↑ *CCL22* ↑	(42, 43)
RAW264.7	Resting condition	Ferumoxytol (2.73 mg/ml, equal to 48.9 mM Fe^2+^), 24 h	TNF-α↑CD86 ↑ IL-10 ↓ CD206↓	(44)
	IFN-γ (20 ng/ml), 24 h	FAC, (0.089 mM Fe^3+^)	IL-1β↓ TNF-α ↓ iNOS ↓	(35)
	Resting condition	Fe^3+^ (ferric citrate, 2.5 mg/ml, 10.2 mM Fe^3+^) or Fe^2+^ (ferrous citrate, 2.5 mg/ml, 10.2 mM Fe^2+^), 2 h	IL-1β↑ TNF-α↑ iNOS ↑	(45)
	LPS (100 ng/ml)+IFN-γ (20 ng/ml) or IL-4 (20 ng/ml)+ IL-13 (20 ng/ml), 24h	FAC (0.05-0.15 mM)	GMFG ↓	(38, 46)
BMDM	Resting condition	FAC (0.25 mM), 4 h	iNOS↑ CCL2↑ IL-1β↑ KLF4↓	(47)
	LPS+IFN-γ or IL-4+ IL-13, 20 ng/ml, 24 h	FAC (0.1 mM)	IL-1β↓ *CCL17*↑ *CCL22* ↑	(42)
	IL-4 (20 ng/ml), overnight	Iron dextran (20 mM) or RBCs (10:1)	CD16/32 ↑ CD206 ↓	(38, 42)

RBCs, Red blood cells; “↑”, increase; “↓”, decrease.

Interestingly, iron supplementation also affects macrophage phenotypes. Increasing endogenous iron induces TNF production in macrophages (38). Besides, exogenous iron supplementation, like ferric ammonium citrate (FAC) (0.25 mM Fe^3+^), ferric citrate (2.5 mg/ml, equal to 10.2 mM Fe^3+^), ferrous citrate (2.5 mg/ml, equal to 10.16 mM Fe^2+^), and Ferumoxytol (Fe_3_H_2_O_4_) (2.73 mg Fe/ml, equal to 48.9 mM Fe^2+^), promotes the expression of M1 markers (e.g., iNOS, TNF-α, and IL-1β), while lowers the expression of M2 markers (e.g., IL-10 and CD206) in RAW264.7 cells (44, 45), bone marrow-derived macrophages (BMDMs) (47), and THP-1 cells (40, 41). In addition to the *in vitro* experiments, some *in vivo* experiments also confirm that iron supplementation enhances pro-inflammatory macrophage polarization (37, 47).

The roles of iron in regulating pro-inflammatory macrophages are not always consistent. In IFN-γ-stimulated RAW264.7 cells, FAC supplementation (25 μg/ml, equal to 0.089 mM Fe^3+^) lowers the expression of M1 markers (e.g., IL-1β, iNOS, and TNF-α) by blocking signal transducer and activator of transcription (STAT)-1 pathway (35). Moreover, supplementation of Fe^2+^ (FeSO_4_, 0~1 mM) reduces LPS/IFN-γ-induced NO synthesis in mouse and rat macrophages (39). The following factors may be conceivably involved in the regulation of the final polarization trends of macrophages: (i) the final iron (Fe^2+^ and/or Fe^3+^) concentrations and different processing time (48); (ii) the macrophage types (e.g., RAW264.7 cells, THP-1 cells, and BMDMs); (iii) the various conditions of stimuli/inducers for macrophage polarization; (iv) pretreatment of iron or treatment of iron during macrophage polarization simultaneously. Hence, it is worth conducting experiments to validate the aforementioned speculations, as well as the genders of experimental animals or other factors associating the various results of macrophage polarization status under iron treatment.

In contrast to classically-activated macrophages, alternatively-activated macrophages are dominated by low Ft and high FPN, and the high heme oxygenase (HO)-1 expression promotes the absorption of heme and the excretion of free iron by alternatively-activated macrophages (49). Therefore, iron homeostasis may be also closely related to the functions of M2-like macrophages, which has been validated in models of diabetes (42), spinal cord injuries (SCI) (38), and cancers (44, 45). For example, tumor-associated macrophages (TAMs) are defined as M2-like macrophages that promote the progression of tumor and are associated with poor prognosis (50–52). In tumor-burden BALB/C mice, ferric citrate (2.5 mg per three day, totally 17.5 mg) injection promotes reactive oxygen species (ROS) production and CD86 expression of tumor infiltrating macrophages, and lowers the volume and weight of H22 hepatoma xenograft (45). Likewise, intravenous injection of ferumoxytol (10 mg Fe/kg) to mice profoundly inhibits the function of M2-like macrophages while enhances the function of M1-like macrophages in tumorous tissues (44). Collectively, these findings suggest that iron negatively regulates M2-like macrophage polarization.

In some situations, iron may promote M2-like macrophage polarization. For example, exogenous iron supplementation (FAC, 0.1 mM) and endogenic iron-rich human dermal fibroblasts extracellular matrix (ECM) both enhance THP-1 cells polarizing into M2 phenotype (42). Similar finding is also observed in BMDMs (42). Interestingly, iron status causes different inflammatory response outcomes (44, 53, 54). For example, iron deprivation lowers the expression of M2 markers, while an iron-rich status (under physiological iron loading condition) favors M2-like macrophage polarization and inhibits LPS-induced inflammatory responses, which might be highly associated with the differential regulation of *Hamp* and *FPN* in the context of various inflammatory conditions (55). These conflicting results suggest that iron might regulate macrophage polarization by affecting different signal pathways based on different models. Therefore, the underlying mechanisms by which iron shapes the macrophage polarization deserve further exploration.

In summary, in most cases (concerning nontumorous and/or tumorous tissues), iron affects macrophage phenotypes by promoting classically-activated macrophage functions [especially high iron addition (>0.1 mM Fe^3+^ and/or >1 mM Fe^2+^)], while decreasing alternatively-activated macrophage functions (**Table 1** **)**. It should be noticed that some effects seen are probably the related stress responses rather than iron-driven macrophage polarization (43). The differences in the direction of macrophage polarization caused by iron deficiency, iron supplementation or overload also reflect that the cellular signaling and/or metabolic pathways underlying iron in shaping macrophage polarization are complex and diverse, and the specific mechanisms remain to be explored.

## Mechanisms Whereby Iron Mediates Macrophage Polarization

### Iron Shapes Macrophage Polarization Through Cellular Signaling Pathways

**Figure 1:**
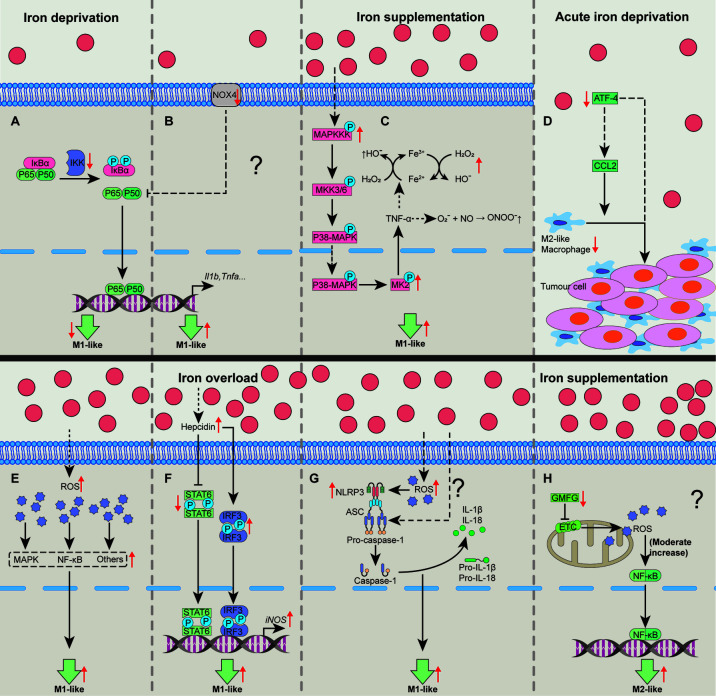
Cellular pathways whereby iron shapes macrophage polarization. Iron deprivation may suppress NF-κB activation to block M1-like macrophage polarization **(A)**. In some special cases, iron deprivation could lower the activity of Nox to promote p65 nuclear translocation and increases NF-κB activity, promoting M1-like macrophage polarization **(B)**. Iron treatment could activate MAPK-p-MK2 pathway to increase TNF-α production to generate a large amount of OH• and ONOO•, promoting M1-like macrophage polarization **(C)**. Acute iron deprivation inhibits ATF4 expression to suppress M2-like macrophage polarization in tumorous tissues **(D)**. Iron treatment may promote M1-like macrophage polarization by inducing the activation of MAPK, NF-κB and other proinflammatory signaling pathways *via* increasing the production of intracellular ROS **(E)**. Iron overload increases hepcidin expression which can inhibit STAT6 activation while promote IRF3 expression to increase iNOS expression in M1-like macrophages **(F)**. Iron can activate NLRP3 inflammasome to promote M1-like macrophage polarization by the generation of ROS **(G)**. Iron might enhance M2-like macrophage polarization through moderately activating of mtROS cascading with NF-κB pathway by down-regulating GMFG level **(H)**. ATF4, activating transcription factor 4; GMFG, glia maturation factor-γ; IRF3, interferon regulatory factor 3; MAPK, mitogen-activated protein kinase; MK2, kinase 2; NF-κB, nuclear factor κB; NLRP3, NOD‐like receptor; Nox, NADPH oxidase; ROS, reactive oxygen species; STAT6, signal transducer and activator of transcription 6. “↑”, increase; “↓”, decrease. Question mark means that the mechanism needs experimental confirmation.

Within multifarious pathways associating with functions of macrophages, NF-κB functions as a main contributor to regulate macrophage polarization (30, 56). Iron deprivation shapes macrophage polarization through NF-κB involving distinct mechanisms. Interestingly, macrophages isolated from iron deficient rats have reduced Nox activity while iron depletion blocks the activation of NF-κB in LPS+TNF-α-activated macrophages (37), implying the suppression of classically-activated macrophage polarization by iron deprivation (**Figure 1A**). However, LPS/IFN-γ-polarized macrophages with NADPH oxidase (Nox) 4 deficiency [a member of Nox family (57)] express more Nox2 to enhance p65 nuclear translocation and NF-κB activity to force pro-inflammatory macrophage polarization (58), indicating that there might be another mechanism involving NF-κB for iron deprivation to affect M1-like macrophage polarization (**Figure 1B**). Here, we speculate that these conflicting results might be associated with the different culture conditions and/or the levels of Nox2; however, the aforementioned process still needs well-designed experimental validation.

Iron also shapes macrophage polarization through mitogen-activated protein kinase (MAPK). Iron supplementation or intracellular iron accumulation increases TNF-α expression (38, 44, 54). Mechanistically, iron accumulation enhances expression of MAPK, leading to the phosphorylation of kinase 2 (MK2) to trigger TNF-α production (38, 59). TNF-α continuously stimulates macrophages to produce a large number of hydroxyl radicals (OH^•^) and peroxynitrite (ONOO^•^) (54, 60). Thus, iron treatment may promote M1-like macrophage polarization through MAPK-p-MK2 axis (**Figure 1C**). Lipocalin-2 (Lcn-2) combined with siderophore-bound iron can be absorbed by macrophages (61). Interestingly, Lcn-2 deficiency reduces M1 polarization in BMDMs (62). Mechanistically, Lcn-2 activates ERK1/2 to shape macrophage towards M1-like phenotype (63). However, whether Lcn-2-mediated intracellular iron accumulation causes M1-like macrophage polarization is still lacking the direct evidence.

Other cellular signaling pathways include ATF4, ROS, and NLRP3 inflammasome. ATF4 expression is correlated with the percentage of M2-like macrophages (64, 65). Notably, acute iron deprivation decreases ATF4 (36), indicating that iron deprivation could block M2-like macrophage polarization (**Figure 1D**). Furthermore, iron treatment may promote M1-like macrophage polarization by increasing the production of intracellular ROS (**Figure 1E**) (38, 45, 66). Iron overload increases hepcidin expression (45), which inhibits STAT6 activation while promotes interferon regulatory factor (IRF)-3 expression (40), resulting in pro-inflammatory macrophage polarization (**Figure 1F**). NLRP3 inflammasome (mainly including NLRP3, ASC, Caspase-1, and IL-1β) functions as a crucial platform for M1-like macrophage polarization (30). Of note, iron overload can activate NLRP3 inflammasome through the generation of ROS, and some unrevealed mechanisms (67–69), thereby enhancing M1-like phenotype (**Figure 1G**).

On the contrary, iron supplementation may also inhibit M1-like polarization by suppressing STAT1 activation (35). Iron supplementation down-regulates actin-regulatory protein glia maturation factor-γ (GMFG), which is negatively associated with the mitochondrial ROS (mtROS) accumulation (46). The moderate increase of mtROS cascading with NF-κB pathway is needed for M2-like macrophage polarization (70); hence, iron supplementation might also shape M2-like macrophage polarization associating with GMFG/mtROS/NF-κB pathway (**Figure 1H**). Therefore, iron supplementation and/or overload affect macrophage polarization depends on various cellular pathways (**Figure 1**). And the possible reasons for these different effects of iron in guiding macrophage polarization are largely related to cellular iron status (e.g., marginal or high iron supplementation) and/or heterogeneity of tissue microenvironment.

### Iron Shapes Macrophage Polarization Through Cellular Metabolism

**Figure 2:**
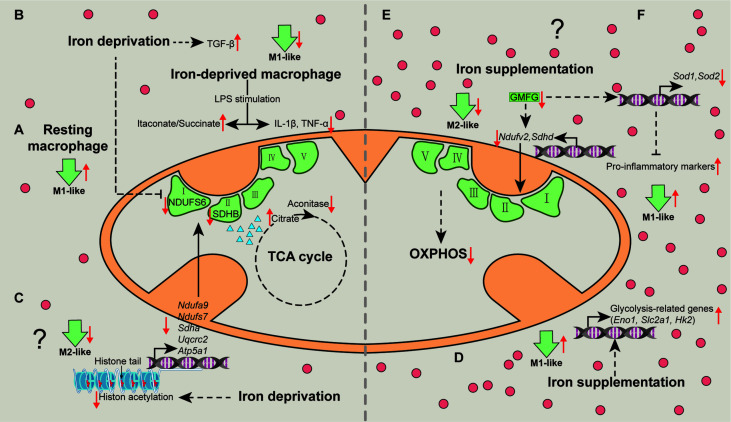
Mechanisms associated with metabolic pathways whereby iron shapes macrophage polarization. Iron deprivation directly inhibits the expression of NDUFS6 and SDHB and blocks the activity of mitochondrial aconitase to cause citrate accumulation, thereby switching resting macrophages towards M1-like macrophages **(A)**. Iron deprivation limits M1 polarization by increasing itaconate:succinate ratio through altering metabolic fluxes and inducing TGF-β signaling pathway **(B)**. Specially, iron deprivation decreases histone acetylation to reduce transcription of the nuclear DNA-encoded OXPHOS genes, suppressing M2-like phenotype **(C)**. Iron-load increases the expressions of glycolysis-related genes to promote glycolysis in macrophages, enhancing M1-like macrophage polarization **(D)**. Iron might restrain M2-like macrophage, while favors M1-like macrophage polarization *via* reducing the expression of NDUFV2 and SDHD **(E)**, and SOD1 and SOD2 through down-regulating GMFG **(F)**, respectively. OXPHOS, oxidative phosphorylation; TGF-β, transforming growth factor-β. “↑”, increase; “↓”, decrease. Question mark means that the mechanism needs experimental confirmation.

The cellular metabolism is highly associated with the functional output of macrophages (31). Unfortunately, there are only several studies concerning the cellular mechanisms whereby iron mediates macrophage polarization. Of note, although acute iron deprivation in human macrophages without pro-inflammatory stimuli (resting macrophages) share the same metabolic switch with pro-inflammatory stimuli (e.g., LPS and/or IFN-γ)-treated macrophages (an induction of glycolysis and fatty acid synthesis, and a repression of OXPHOS), the underlying mechanisms differ (36). Mechanistically, iron deprivation directly inhibits the mRNA expression and protein abundance of respiratory chain Fe-S enzymes (including NDUFS6 and SDHB) and blocks the activity of mitochondrial aconitase to cause citrate accumulation (36), thereby potentiating M1-like phenotype (**Figure 2A**); nevertheless, LPS-induced M1-like macrophage polarization is limited in iron-deprived human macrophages due to the increase of itaconate:succinate ratio (**Figure 2B**) (36). Prolyl hydroxylase (PHD), as a key factor in the metabolic pathways, is responsible for M1-like macrophage polarization *via* post-translational modification of IKKβ and stabilization of HIF‐1α (71–74). Notably, lack of iron inhibits PHD proteins, plausibly explaining how iron deprivation facilitates M1-like macrophage polarization in some specific situation (e.g., acute iron deprivation). Interestingly, iron deprivation also results in reduced transcription of the nuclear DNA-encoded OXPHOS genes [e.g., *Ndufa9* and *Ndufs7* (complex I), *Sdha* (complex II), *Uqcrc2* (complex III) and *Atp5a1* (complex V)] *via* modulating nuclear epigenome (chiefly decreasing histone acetylation) (75), suggesting that iron depletion may suppress M2-like phenotype (**Figure 2C**). It is mentionable that the changes in cellular metabolism are likely the outcomes of epigenetic regulation; hence, whether and how iron deprivation facilitates resting and/or pro-inflammatory-activated macrophages towards M1-like phenotype through the decreased acetylation of OXPHOS-related genes still needs experimental validation.

As for iron supplementation, it has been demonstrated that iron-load promotes glycolysis in macrophages, manifested by increased expression of glycolysis-related genes, such as *Eno1*, *Slc2a1*, and *Hk2* (76), indicating that iron promotes M1-like macrophage polarization through glycolysis (**Figure 2D**). Considering that iron-load down-regulates GMFG (46), and that blockade of GMFG reduces the expression of NDUFV2 (complex І), SDHD (complex II), and SOD (77), iron might restrain M2-like macrophage polarization *via* GMFG-OXPHOS pathway (**Figure 2E**) and/or favor M1-like macrophage polarization through GMFG-SOD signaling (**Figure 2F**). Overall, iron mainly shapes activated macrophages polarize into pro-inflammatory phenotype *via* targeting cellular metabolism (**Figure 2**).

### Iron Shapes Macrophage Polarization Through Epigenetic Regulation/Modification

**Figure 3:**
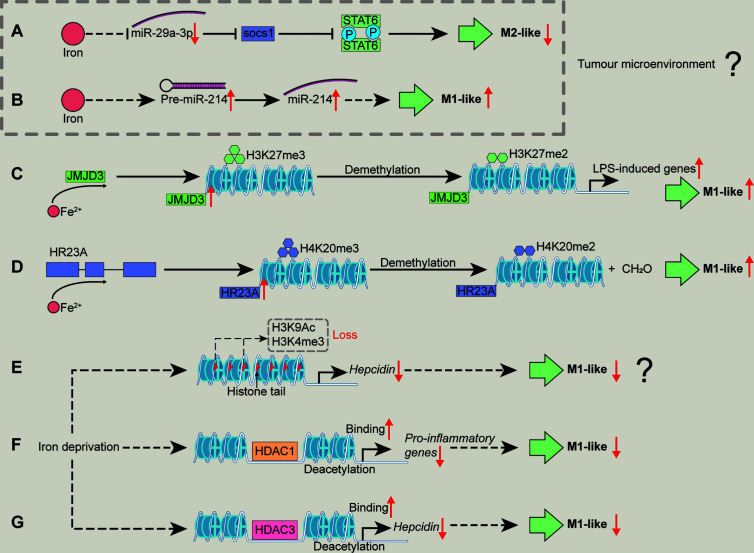
Mechanisms associated with epigenetic regulation/modification whereby iron shapes macrophage polarization. Iron supplementation may reduce miR-29a expression to block the activation of SOCS1/STAT6 signal pathway, inhibiting TAM polarization **(A)**. Iron could promote M1-like macrophage polarization by increasing miR-214 expression **(B)**. Iron could increase the enzyme activity of JMJD3 to demethylate H3K27me3, relieving the transcriptional silencing of LPS-induced genes and facilitating M1-like macrophage polarization **(C)**. Iron might promote macrophages toward pro-inflammatory phenotype through demethylating H4K20me3 by enhancing the enzyme activity of HR23A **(D)**. Iron deficiency might cause the loss of H3K9Ac and H3K4me3 to reduce hepcidin expression, inhibiting M1-like macrophage polarization **(E)**. Iron deficiency inhibits M1 activation (e.g., pro-inflammatory responses) possibly through increasing HDAC1 binding to the related genes **(F)**. Iron deficiency inhibits M1-like phenotype by lowering hepcidin expression *via* enhancing HDAC3 binds chromatin at the hepcidin locus **(G)**. H3K27me3, histone 3 lysine 27 trimethylation; H4K20me3, histone 4 lysine 20 trimethylation; HDAC, histone deacetylase; SOCS1, suppressor of cytokine signaling 1. “↑”, increase; “↓”, decrease. Question mark means that the mechanism needs experimental confirmation.

Generally, epigenetic regulation/modification includes small RNA, DNA modification (methylation), histone modification, and chromatin remodeling. Epigenetic regulation/modification causes chromosome-bound, heritable changes to gene expression that are not dependent on changes to DNA sequence (78), thus becoming a key platform for macrophage reprogramming (79–81). In this section, we summarize how iron shapes macrophage phenotypes through the interactions between iron and several epigenetic regulators (**Figure 3**).

### MiRNAs

MicroRNAs (miRNAs) are a series of non-coding RNA involved in regulating gene expression. With the establishment of miRNAs expression profiles associated with the polarized human and mouse macrophages, it is not surprising that miRNAs function in controlling macrophage polarization (82). The expression of miR-141-3p or miR-152 is highly up-regulated in pro-inflammatory macrophages; while the expression of miR-124 is increased in anti-inflammatory macrophages (82). MiR141/200c could inhibit adenosine 5’-monophosphate (AMP)-activated protein kinase (AMPK) phosphorylation to block the activation of peroxisome proliferators-activated receptor (PPAR)-α, leading to an impairment of fatty acid β-oxidation in macrophages (83). Therefore, miR-141 favors M1-like phenotype (83). Interestingly, the anti-inflammatory effects of miR152 appear to be inconsistent with their up-regulation in M1-like macrophages. MiR-152 directly targets to Kruppel-like factor 5 (KLF5) to inhibit M1-like macrophage polarization (84). MiR-124 favors M2-like phenotype by inhibiting LPS-induced STAT3 phosphorylation in LPS-exposed RAW264.7 cells (85).

Of note, some miRNAs associating with macrophage polarization are supposed to regulate the expression of iron-regulated genes. For example, miR-124 modulates the stability of TFR1 mRNA by targeting iron-responsive element (IRE)-B (86); miR-152 negatively regulates TFR1 at post-transcriptional level (87); miR-141-3p regulates TFR1 mRNA degradation by directly targeting 3’-untranslated region (UTR) of TFR1 (86) or not (88, 89). Insofar, it is not surprising that iron shapes macrophage polarization through miRNAs.

MiR-29a-3p enhances the polarization of M2-like macrophages by activating suppressor of cytokine signaling (SOCS)-1/STAT6 signal pathway (90). Considering iron negatively regulates miR-29a (91), iron supplementation may reduce miR-29a expression to inhibit M2-like macrophage polarization (**Figure 3A**). Moreover, miR-214 is related to M1 polarization (92–94) and iron saccharate promotes miR-214 transcription (95); thus, it is likely that iron could promote M1-like macrophage polarization by increasing miR-214 expression (**Figure 3B**). However, these observations about iron-mediated the expression of miRNAs are achieved by TAM; therefore, whether iron shapes the polarization of conventional macrophages *via* miRNAs needs further investigations.

### DNA Methylation

DNA methylation is associated with the transcriptional inhibition *via* DNA methyltransferases (DNMTs, including DNMT1, DNMT2, DNMT3A, and DNMT3B) (96). Up to now, DNA methylation in modulating M1 activation is still in its infancy. Only DNMT1 has been found to inhibit the expression of SOCS1 (which functions as a negative regulator of cytokine signals, including STAT1) through hypermethylation, thereby enhancing M1-like macrophage polarization (97). Interestingly, different concentrations of exogenous iron (Fe^3+^, 0.0625~0.25 mM) directly decrease the enzyme activity of purified DNMT *in vitro* (98). In addition, as mentioned above, FAC supplementation (~0.089 mM Fe^3+^) suppresses M1-like macrophage polarization by blocking STAT1 pathway in IFN-γ-stimulated RAW264.7 cells (35); thus, it remains to know whether in some special cases, iron blocks STAT1 pathway through regulating DNA methylation.

### Histone Modification

Histone 4 lysine 20 trimethylation (H4K20me3), H3K9me3, and H3K27me3 restrain inflammatory cytokine gene transcription (e.g., IL-12 p40) in the absence of TLR signaling to keep macrophages in a “poised” state (32). Notably, these epigenetic “brakes” related to transcriptional silencing could be released by using demethylases in macrophages followed by inflammatory stimulation (e.g., TLR ligands) (32). Indeed, these modifiers involved in the regulation of macrophage polarization are also inextricably linked to iron. LPS-triggered NF-κB activation promotes JMJD3 (specially demethylates H3K27me3) binding to the promoter regions of LPS-induced genes in macrophages (99, 100). Notably, the enzyme activities of JumonjiC histone demethylases (including JMJD3) depend on 2-oxoglutarate and iron (II); thus, it is likely that iron could increase the enzyme activity of JMJD3 to demethylate H3K27me3, relieving the transcriptional silencing of LPS-induced genes and facilitating M1-like macrophage polarization (**Figure 3C**). Moreover, HR23A is a histone H4K20 demethylase that specifically demethylates H4K20me1/2/3 to produce formaldehyde, and iron (II) is required as cofactor for the enzyme activity of HR23A (101). Herein, we envision that iron might promote macrophages towards pro-inflammatory phenotype through demethylating H4K20me3 by enhancing the enzyme activity of HR23A (**Figure 3D**). Moreover, it has been shown that ten-eleven translocation family proteins (Tets) [which are iron (II) and α-ketoglutarate (α-KG)-dependent demethylases (102–104)] promote (by Tet1) (105) or restrain (through Tet2) (106, 107) pro-inflammatory responses in macrophages through unique mechanisms; thus, iron might also shape M1-like macrophage polarization through regulating Tet activity (Figure not shown).

Indeed, after removal of “negative histone markers” (which could inhibit gene expression, e.g., H4K20me3, H3K9me3, and H3K27me3) of inflammatory genes, “positive histone markers” (which could promote gene expression, including H3K4me3, H3K9Ac, and H3K27Ac) increase, that could be enriched in the promoter region of *Cxcl10* (M1 marker gene) (32). Notably, iron deficiency suppresses hepcidin expression involving reversible loss of H3K9Ac and H3K4me3 at the hepcidin locus (108). Given that hepcidin supplementation promotes M1-like polarization (40), we speculate that iron deficiency might cause the loss of H3K9Ac and H3K4me3 to reduce hepcidin expression, inhibiting M1-like macrophage polarization (**Figure 3E**). Nevertheless, the above hypothesis still requires the convincing experimental evidence.

It should be noticed that HDACs highly regulate M1 activation; and LPS stimulation affect almost all classes of HDACs, thereby controlling the expression of inflammatory genes. For instance, HDAC 2/3/6/9 positively regulate M1 polarization, whereas HDAC 1/4/5 have repressive effects (32, 109). It has been found that the initial decrease in HDAC1 is responsible for IL-8 induction during *L. pneumophila* infection (110). Fetal iron deficiency increases HDAC1 binding to its target gene promoter (111). Therefore, iron deficiency inhibits M1 activation (e.g., pro-inflammatory responses) possibly through increasing HDAC1 binding to the related genes (**Figure 3F**). Besides, HDAC3 depletion leads to the loss of basic expression of IFN-β in macrophages, which affects subsequent STAT1-dependent gene expression and thus inhibits inflammatory activation (109). Intriguingly, during iron deficiency, RGFP966 (the HDAC3 inhibitor) counteracts hepcidin suppression (108). As mentioned above, hepcidin could promote M1-like macrophage polarization (40); thus, it is possible that iron deficiency inhibits M1-like phenotype by lowering hepcidin expression *via* enhancing HDAC3 binds chromatin at the hepcidin locus (**Figure 3G**). Besides, HDAC1 reduces expression of hepcidin by interacting with SMAD4 rather than deacetylation of SMAD4 or histone-H3 on the hepcidin promoter (112); therefore, it is interesting to further investigate whether iron deficiency directs pro-inflammatory macrophage polarization that is independent of the effect of deacetylation of HDAC1.

Sirtuin 2 (SIRT2) depletion results in a decrease in cellular iron levels; mechanistically, SIRT2 deacetylates nuclear factor erythroid-derived 2-related factor 2 (NRF2) on K506 and K508 to reduce FPN1 expression, leading to the decreased cellular iron export (113). After TNF-α stimulation, Sirt2^-/-^ mouse embryonic fibroblasts (MEFs) cells become highly acetylated, resulting in increased expression of NF-κB target genes, including *Mpa2l*, *Cxcl5*, *Ip10*, and *Il6* (114). Reciprocally, whether iron status shapes M1 polarization as an outcome of the effect of SIRT2/NRF2 is still unknown. Importantly, a large number of iron deprivation-related histone acetylation and methylation modifications have been found in iron chelator-treated myoblasts cells (75), thus it would be very interesting to explore whether these epigenetic modifications could participate in regulating macrophage polarization in the context of iron deprivation.

Indeed, chromatin remodeling occurs through the ATP-dependent SWI/SNF complex during M1 activation (115), and SWI/SNF directly represses iron transport-related genes (116); therefore, it is of great interest to investigate whether iron shapes M1 polarization involving chromatin remodeling in the future.

## Iron in Macrophage-Associated Diseases

Notably, defects in macrophage phenotypic switch, such as the prolonged activation of a pro-inflammatory property in macrophages, are associated with various macrophage-associated diseases (117). Thus, the significant role of iron in dictating macrophage polarization suggests that iron has potential for affecting the progression of macrophage-associated diseases.

### Nonalcoholic Fatty Liver Disease

Liver is the main organ for systemic iron regulation and storage, and the increase of iron in the liver may cause oxidative damage, lipid peroxidation, and even cell death (118). Nonalcoholic fatty liver disease (NAFLD) is one of the most common chronic liver diseases, including steatosis, nonalcoholic steatohepatitis (NASH), cirrhosis, and hepatocellular carcinoma (119).

In patients with NAFLD, iron deposits in different liver cells [including reticuloendothelial system (RES)]. Of note, iron deposits in RES could aggravate clinical symptoms of patients (120) due to the accumulation of inflammation-induced iron in Kupffer cells *via* IL-6/hepcidin pathway (121, 122). Simultaneously, the accumulation of iron in RES also promotes the production of ROS, inducing lipid peroxidation, apoptosis and/or necrosis of Kupffer cells (123). Similarly, in high-fat-induced NASH model, dietary iron supplementation induces iron accumulation in sinusoidal macrophages as well as high level of TNF-α in liver, aggravating disease symptoms (124). However, in thioacetamide-induced cirrhosis model, after fed with high-iron diet, although macrophages are recruited in the liver sinusoid, the number of macrophages infiltration within the hepatic lesions is reduced, thereby the hepatocellular injury could be attenuated, which is probably due to iron alters the apoptotic pathway (125).

### Atherosclerosis

Atherosclerosis is a common vascular disease with the formation of plaques or lesions in the arterial wall (126). Macrophages are the main inflammatory cells in atherosclerotic plaques. During atherogenesis, circulating monocyte-derived macrophages could be recruited to vascular endothelium and form lipid-laden foam cells (one of the main characteristics of atherosclerosis) (127). Indeed, according to the “iron hypothesis”, overloaded iron generates a large amount of ROS and promotes the lipid-peroxidation, inducing fatty deposits and inflammatory cell infiltration (128). During atherogenesis, high levels of iron in circulation promotes the recruitment of monocyte-derived macrophages to vascular endothelium, and excessive iron stimulates M1-like macrophage polarization in plaques, thereby facilitating the formation of foam cells (129–131). Hepcidin level is supposed to be a major determinant of the level of iron retained within macrophages (20, 132). Of note, impaired clearance of iron from plaque macrophages caused by high inflammation-induced levels of hepcidin might be associated with atherogenesis (133). Thus, it may be possible to prevent atherosclerosis by reducing iron retention in the plaque macrophages through decreasing hepcidin concentration (134, 135).

### Cancer/Tumor

TAMs are M2-like macrophages related to the progression and poor prognosis of tumor (136–138). Iron-containing M2 macrophage conditioned medium contributes to the growth of human renal cell carcinoma cells (RCC10) or mouse NIH-3T3 cells; and chelation of iron by desferrioxamine (DFO) supplementation reduces cancer cell proliferation (139). Moreover, co-culture of TAM and melanoma cells exhibit coexpression of HO-1 and CD163, suggesting that tumor cells-derived M2 macrophages promote heme metabolism (140). These studies indicate that M2 macrophages metabolize iron and delivery iron to tumor cells. Indeed, iron chelation therapy for cancer is used clinically. In addition to DFO, there are several other drugs (like deferiprone, deferasirox, and curcumin) are applied for slowing down cancer progression (141). Interestingly, except for iron chelation-based cancer therapy, iron supplementation (e.g., iron oxide nanoparticles) also shows highly anti-tumor potential. For example, ferumoxytol inhibits tumor growth and metastases that are closely associated with M1 polarization and cancer cell apoptosis induced by Fenton reaction (44).

Collectively, these interesting findings suggest the various roles of iron in macrophage-associated diseases through altering macrophage responses. Together with the other macrophage-associated diseases summarized in **Table 2**, the specific pathways transmitting the effects of iron on these diseases need to be fully understood.

**Table 2:** Iron in other macrophage-associated diseases.

Diseases	Iron-related clinical symptoms	Macrophage responses	Refs
Hereditary Hemochromatosis	Hepatocyte iron overload	FPN mutation blocks macrophages iron export and causes cellular iron overload.	(142, 143)
Multiple Sclerosis	Dysregulated brain iron and iron presents around plaques	Oligodendrocytes release massive iron and facilitate the expression of NADPH oxidase in microglia, impairing mitochondrial functions.	(144–146)
Chronic Kidney Disease	The contents of circulating iron in the body are decreased	Inflammation leads to iron chelation in macrophages, limiting erythropoiesis.	(147, 148)

## Concluding Remarks

Iron is an essential metal that fine-tunes a series of physiological functions, including macrophage-dependent responses. In this review, we summarize how iron shapes macrophage polarization. Mechanistically, iron modulates macrophage polarization *via* cellular signaling pathways (e.g., MAPK, NF-κB, and STATs), cellular metabolism (mainly targeting OXPHOS), and epigenetic regulation (e.g., miRNAs, DNMT1, JMJD3, H3K4me3, H4K20me3, and HDAC1/3). Other pathways discussed in this review seem to additionally contribute; however, these require a broader experimental confirmation. It has been well documented that α-KG produced by glutaminolysis is responsible for JMJD3-dependent demethylation of H3K27 on the promoter of M2‐specific marker genes (149). Thus, it would be very interesting to study whether iron shapes macrophage polarization (M2 polarization in particular) through the correlation of epigenetic and metabolic reprogramming. As we mentioned above, iron may have various roles in regulating macrophage polarization based on different types of macrophages, therefore, it is also meaningful to conduct comparative studies on the effects of iron on polarization of macrophage with different origins (embryonic origin compared with monocyte derivation), tissues (e.g., osteoclasts from bone and alveolar macrophage from lung), or even species (murine and human).

Mitophagy activation or inhibition fine-tunes macrophage polarization (150). Interestingly, iron affects mitophagy through diverse pathways (including mtROS and MAPK) (151, 152). However, the exact mechanisms whereby iron targets mitophagy to switch macrophage polarization should still be revealed with some reservation. Furthermore, mitochondrial dynamics (fission and fusion) is intertwined with macrophage polarization. It has been demonstrated that iron chelation selectively increases mitochondrial fusion protein (Mfn2) expression in neuronal HT22 cells (153), while iron overload induces dephosphorylation of dynamin-related protein (Drp)-1 S637 loci and activation of Drp1 in hippocampal HT-22 neuronal cells (154) as well as promotes mitochondrial fragmentation in mesenchymal stromal cells (MSC) by activating the AMPK/mitochondrial fission factor (MFF)/Drp1 pathway (155). Nevertheless, a broader basis concerning cell types is needed to allow generalizations and robust conclusions on iron in directing macrophage polarization. More recently, it has found that endogenous and/or exogenous iron-induced iron overload can trigger macrophage ferroptosis (156, 157). Macrophage under hypoxia condition undergoes ferroptosis (158, 159), thus whether iron shapes macrophage polarization (chiefly M1 polarization) through affecting ferroptosis should also be taken into consideration. More importantly, it is urgent to dig out the intrinsic relationship between ferroptosis, macrophage phenotype, and cancer progression (160).

## Author Contributions

YX designed the review article. YX, YL, and XW wrote the review article. YX, XM, and MY revised the review article. YL, XW, QZ, and SC helped with designing figures and finding relevant literatures. YX, XM, and MY approved the final manuscript. All authors contributed to the article and approved the submitted version.

## Funding

This work was supported by the National Natural Science Foundation of China (31902200), Special fund for scientific innovation strategy-construction of high level Academy of Agriculture Science (R2017PY-JG001, R2020YJ-YB2002), and Agricultural competitive industry discipline team building project of Guangdong Academy of Agricultural Sciences (202118TD).

## Conflict of Interest

The authors declare that the research was conducted in the absence of any commercial or financial relationships that could be construed as a potential conflict of interest.
